# The Moderating Effects of Race and Ethnicity on a Motivational Interviewing Intervention for Improving Physical Health Among Patients With Peripheral Artery Disease: Secondary Analysis of a Pilot Randomized Trial

**DOI:** 10.2196/82926

**Published:** 2026-10-15

**Authors:** Yiliang Zhu, Tracie Collins

**Affiliations:** 1 School of Medicine University of New Mexico Albuquerque, NM United States; 2 College of Population Health University of New Mexico Albuquerque, NM United States

**Keywords:** peripheral artery disease, motivational interviewing, health disparities, social determinants of health, effect modification, intervention heterogeneity, 6-minute walk, weight loss

## Abstract

**Background:**

In a pilot randomized trial, face-to-face or telephone motivational interviewing (MI) was more efficacious than a smartphone app after 3 months for increasing 6-minute walk distance (primary outcome) and reducing body weight (secondary outcome) in patients with peripheral artery disease (PAD). Little is known about intervention heterogeneity related to social determinants of health (SDOH).

**Objective:**

This study aimed to explore whether SDOH are associated with the MI intervention effects on walking distance and weight loss and generate intervention heterogeneity hypotheses.

**Methods:**

Community dwellers in Wichita, Kansas, were recruited through community outlets and screened for PAD. Consented participants were randomly assigned to MI (n=16) or an app (n=13). Survey instruments were used to measure SDOH, demographics, and adverse childhood experiences, which were assessed for their associations with 6-minute walk distance change and weight loss. Difference-in-differences (DiD) comparisons of median changes between SDOH strata were conducted using Wilcoxon and Kruskal-Wallis nonparametric tests.

**Results:**

For 6-minute walk distance, pretest-posttest changes were significantly greater among participants of “other” race and ethnicity than among non-Hispanic White participants (DiD=44.0 m, 95% CI 19.2-164.2; *P*=.004), and no DiDs for other SDOH were significant. Significant median pretest-posttest increases in walking distance were observed in those with a college or higher educational attainment (48.8 m, 95% CI 28.0-61.4), unmarried individuals (40.4 m, 95% CI 0.5-56.0), those living with family (55.7 m, 95% CI 30.5-199.5), those who talked to and saw family or friends 3 or more times a week (46.4 m, 95% CI 17.2-69.4), and those without unmet daily needs (46.5 m, 95% CI 7.6-67.1). For weight loss, we did not observe significant DiDs with any SDOH. However, a significant median weight loss was observed in every SDOH stratum where we observed a significant increase in 6-minute walk distance. Significant weight loss was also observed in 5 additional SDOH strata: male individuals, household income below US $30,000, never served in military, employed status, and no stress. The improvements in weight loss and walking distance were highly consistent in both direction and size across SDOH strata.

**Conclusions:**

This pilot study was the first to explore potential SDOH-related intervention effect modification and heterogeneity. The significant association between walking distance and race and ethnicity suggested a potential effect modification. Significant pretest-posttest improvements in both outcomes were found in the same SDOH strata. The highly consistent evidence across both outcomes and all SDOH strata provided confidence in potential intervention efficacy and heterogeneity despite the small sample size. Our findings were consistent with established associations between SDOH and general functions in patients with PAD, suggesting likely mediation pathways through health literacy and social support. Future interventions should consider heterogeneity to target socially disadvantaged patients with PAD to bridge disparities and maximize benefits.

**Trial Registration:**

ClinicalTrials.gov NCT03694652; https://clinicaltrials.gov/ct2/show/NCT03694652

**International Registered Report Identifier (IRRID):**

RR2-10.2196/30295

## Introduction

Peripheral artery disease (PAD) is a disabling illness that affects approximately 5% of adults in their late 40s to 18% of adults in their late 80s in high-income nations [1]. PAD is estimated to affect more than 12 million Americans and 200 million people worldwide [2]. In the United States, an estimated 19%, 22%, and 30% of non-Hispanic White, Hispanic, and non-Hispanic Black persons, respectively, will experience lifetime incident PAD by 80 years of age [3]. Symptomatic PAD (objective evidence of PAD with exertional leg symptoms) has a profound impact on walking ability, including a reduction in walking distance, speed, and stair-climbing ability [4]. These outcomes, in turn, are predictive of a greater mortality risk [4]. Physical inactivity and obesity, common among adults in the United States, are significant risk factors for PAD progression and its adverse outcomes [5]. Prior trials have demonstrated the efficacy of walking therapy in improving physical and biological functions as well as quality of life in patients with symptomatic PAD [6,7]. To date, successful walking therapy interventions have largely been delivered through face-to-face and/or telephone counseling [8-11]. The widespread adoption of walking interventions has been hindered by their labor-intensive staffing requirements. As smartphone use permeates all ages, cultures, and socioeconomic backgrounds, the use of a smartphone app for walking interventions may avoid this limitation.

Collins et al [12] conducted a randomized pilot trial among patients with overweight or obesity and with PAD to assess the comparative effectiveness of a novel smartphone eHealth app vs a motivational interviewing (MI) intervention for increasing walking distance and promoting weight loss at 3 months after the intervention. Details of the study, including the inclusion and exclusion criteria, participant recruitment, trial procedures, and the intervention content, are reported in the paper by Collins et al [12]. The authors reported median increases of 40.5 and 8 m for the MI and app groups, respectively, in the primary outcome of 6-minute walk distance (difference-in-differences, DiD; *P*=.03), greatly exceeding the minimal clinically important difference of 8 m (small) or 20 m (large) [13]. The authors also reported median weight loss (a secondary outcome) of 10.1 and 2.3 lb for the MI and app groups, respectively (DiD *P*=.006). However, Collins et al [12] did not report in-depth analyses of potential differential effects of the interventions between subgroups of participants by their demographics and social determinants of health (SDOH). To our knowledge, there is a dearth of literature on this topic, but it is crucial to inform future studies and programs. Therefore, we conducted a secondary analysis of this pilot trial to investigate intervention heterogeneity by estimating the potential moderation effect of SDOH and other participant characteristics. The primary goal was to generate evidence for new hypotheses concerning intervention heterogeneity.

## Methods

### Study Setting, Sample, and Design

Briefly, the original study [12] was a 2-arm randomized trial to compare the effectiveness of a smartphone app and an MI intervention for increasing walk distance and promoting weight loss. Study participants were recruited from multiple settings, including the University of Kansas hospital system, unaffiliated health centers, and community-based clinics. Area clinicians and administrators distributed flyers and postal mails to eligible patients. Inclusion criteria were age of 50 years or above, overweight or obesity (BMI>27 kg/m^2^), and symptomatic PAD—leg symptoms were captured via a survey, and PAD was confirmed through an ankle-brachial index (the ratio between the systolic blood pressure in the ankle to that in the arm) below 0.9. Individuals were excluded if they exhibited any of the following conditions: intolerance to fruits, vegetables, or fiber or a low-fat diet; restricted water intake; pregnancy; prior major ischemia or critical leg ischemia; use of 24-hour supplemental oxygen; myocardial infarction within the previous 3 months; inability to walk for exercise; or currently walking at least 3 days per week for at least 30 minutes each day.

The original study aimed to recruit 13 participants per arm to reach 80% statistical power to detect a difference of 40 m between the 2 arms. The hypothesized difference between the 2 intervention arms was much greater than published minimal clinically important differences, resulting in the smaller planned sample size. Twenty-nine patients were actually recruited, consented, and completed the study, with 16 randomized to MI and 13 randomized to the app. A post hoc power analysis based on observed sample means and SDs suggested 83% power with this sample size. We note that the original study was not powered to detect intervention heterogeneity or for subgroup analyses.

In the MI arm, participants attended an initial 1-hour face-to-face session followed by 4 20-minute telephone calls: at week 2 and week 4, month 2 and month 3. During each counseling session, the counselor assessed participant willingness to walk for exercise using the Patient-Centered Assessment and Counseling for Exercise survey [14,15]. The counselor queried patients about the importance of exercising and their current motivation to increase walking exercise. The counselor concluded each session by offering patients the option to set a walking goal for the following 2 weeks.

The mobile app had a walking component and a nutritional component and was designed to allow users to set goals for exercise and dietary modifications. There were 6 dietary modification topics specific to each patient. For each topic, the patient was prompted to complete a self-assessment over a 2-week period. The app allowed users to input walking plans, track walking intervals, and record episodic pain. Several safeguards were installed on the app to provide patients with continuous support and resources. The app provided a reporting menu for tracking progress. It automatically sent prompts to encourage its use for diet and exercise management. Participant data were encrypted and saved locally to the phone and were securely sent to an online backup server via the internet. An exit survey was deployed to evaluate participants’ experience and app acceptability, including the number of counseling sessions, frequency of app use, the extent to which the intervention motivated them to exercise and eat healthier, and satisfaction. Data were deidentified before statistical analysis.

A more detailed description of the design, implementation, and analysis of the trial is provided in Multimedia Appendix 1.

### Potential Moderators: SDOH

“Social determinants of health (SDOH) are the conditions in the environments where people are born, live, learn, work, play, worship, and age that affect a wide range of health, functioning, and quality-of-life outcomes and risks” [14]. SDOH can be grouped into 5 domains: economic stability, education access and quality, health care access and quality, neighborhood and built environment, and social and community context [14]. The study data included a number of measures in each domain. Specifically, we used employment status and household income for economic stability; educational attainment for education access and quality; health insurance status and stress for health care access and quality; and gender, ethnicity and race, marital status, living with a family member, any unmet needs or concerns, seeing and talking to family and close friends, and adverse childhood experiences (ACEs) for social and community context. “Any unmet needs or concerns” was a composite score of food, clothing, utilities, transportation, medicine, phone access, and other needs that touched on multiple domains. Multiple instruments were used to collect data related to SDOH and demographics.

### Survey Instruments

#### Protocol for Responding to and Assessing Patients’ Assets, Risks, and Experiences

Much of the SDOH-related data were collected through the Protocol for Responding to and Assessing Patients’ Assets, Risks, and Experiences (PRAPARE) survey [15]. PRAPARE items that were proxies of SDOH included ever being discharged from military service, living alone or with family members, employment status, health insurance status, having any unmet needs or concerns in the previous year regarding 7 items (clothing, utilities, childcare, medicine or health care, phone access, transportation, and “other”), frequency of seeing or talking to people close to them, and stress. Stress is a key pathway through which SDOH may impact health [16]; thus, it is a mediator of SDOH. PRAPARE items that received uniform responses from the participants were excluded from this analysis due to little variation, including any farming income, having housing, worry about losing their house, spending more than 2 days in a row in the previous year in jail, being a refugee, safety of where they live, and being afraid of their partner.

#### ACEs

The ACEs Questionnaire was used to assess the occurrence of potentially traumatic events in the first 18 years of life that might have an impact on both proximal health and distal later-life stressors, contributing to outcomes such as premature death and increased risk of chronic diseases [17,18]. Each of the 10 ACE items asks whether there was childhood exposure to a specific type of adverse experience. We used the total number of ACEs to define 2 ordinal categories: 0 to 1 ACE and 2 or more ACEs.

#### Participant Characteristics

The Lifestyle and Clinical Survey [19] was used to collect participant characteristics: gender, ethnicity and race, marital status, educational attainment, and annual household income.

### Statistical Analysis

We analyzed both the primary and secondary outcomes for the MI arm only because Collins et al [12] found significant pretest-posttest effects in this arm but not in the app arm. Our statistical analyses were anchored on the DiD approach [20] for associations between SDOH strata and the MI intervention because SDOH were not targeted for randomization similar to an observational study. We first stratified study participants by a SDOH variable into 2 strata when feasible to avoid further segmentation of the small study sample. We calculated patient-level pretest-posttest changes in outcomes, then estimated the median pretest-posttest changes (location shifts) within each SDOH stratum using the nonparametric Wilcoxon test and associated exact 95% CIs [21]. A 95% CI that did not include 0 would suggest intervention efficacy in that stratum. We then evaluated differences (ie, DiD) between the medians across the SDOH strata using the Wilcoxon (2 strata) or Kruskal-Wallis (>2 strata) tests [21] alongside 95% CIs. A nonzero DiD would represent a significant association between SDOH and intervention effects, suggesting potential intervention heterogeneity across the strata [22]. We adjusted *P* values of DiD comparisons using false discovery rate correction [23] to maintain the familywise type I error rate at the nominal level (eg, 0.05) for multiple group comparisons. We also used false coverage rate [24] to adjust for multiplicity of DiD CIs as applicable. Our DiD analysis was per treatment, in which patients with missing follow-up outcome data were excluded.

We used the R package *coin* (R Foundation for Statistical Computing) for the Wilcoxon and Kruskal-Wallis tests to produce exact *P* values (Wilcoxon test) and simulation-based approximate *P* values (Kruskal-Wallis test). The DiD parameter includes the interaction term between time (from before to after the intervention) and a dichotomous SDOH under a linear model as a special case. While they faced multiplicity in group comparisons, our DiD analyses circumvented a limitation of the usual multiple regression models with imbalanced and small group sizes, which cannot accommodate multiple covariates and interactions [20].

### Ethical Considerations

The study was registered at ClinicalTrials.gov (NCT03694652). The University of Kansas Medical Center Institutional Review Board approved this study (19-620). Participants gave informed consent before randomization. Participants received US $25, US $30, and US $50 at screening, randomization and baseline, and the 3-month follow-up, respectively. Participant data were saved in a secure location behind a firewall at the University of Kansas Medical Center and were password protected. Data were deidentified for statistical analysis.

## Results

### Participant Characteristics

Of the 16 patients assigned to the MI arm, 2 did not complete the 3-month follow-up. Our per-treatment analysis included 5 male and 9 female participants; 9 non-Hispanic White participants and 5 participants of other races and ethnicities combined; 2 currently married (not separated) and 12 widowed, divorced, or unmarried participants; 5 participants with an educational attainment of college or higher and 9 participants with a level of lower than college; and 7 participants with an annual household income below US $30,000 and 7 participants with an annual household income of US $30,000 or higher.

### Primary Outcome: 6-Minute Walk Distance

For each SDOH, we first reported within-stratum medians in 6-minute walk distance at baseline and at 3 months after the MI intervention and then the within-stratum median pretest-posttest difference and its 95% CI (Table 1). For the SDOH with only 2 strata, we report DiD between the 2 strata in the last column of Table 1, *P* values, and 95% CIs. Imbalances in stratum size and variations in the outcome between strata were visible and common. When a stratum had a size of less than 5, the 95% confidence level might not be achieved for median pretest-posttest changes because the number of possible permutations was limited by the small stratum size. Still, the 95% confidence level might be achieved for the DiD in that case if the other stratum was adequately larger to allow for a sufficient number of permutations. While such a 95% CI and associated test for DiD remain valid, caution is warranted because the DiD results inherited the uncertainty arising from the smaller stratum that did not have a valid CI and the true pretest-posttest difference is uncertain. It should be noted that the median of participants’ pretest-posttest differences is not numerically the same as the difference between the preintervention and postintervention medians when the data deviate from a symmetrical distribution. Below, we illustrate 4 distinct cases of DiD.

At baseline, female participants had a shorter median 6-minute walk distance than male participants (277.8 vs 339.5 m); pretest-posttest median changes were 40.5 and 43.2 m, respectively (Table 1), both twice the threshold for large and clinically meaningful improvement of 20 m [11]. However, these improvements were not statistically significant, and neither was the DiD, suggesting a lack of statistical evidence of intervention efficacy or effect modification by sex, possibly due to the small sample size.

For race and ethnicity, we observed a baseline median walk distance of 309.7 m for non-Hispanic White participants (n=9) and 243.8 m for all other race and ethnicity groups combined (n=5; Table 1). The median pretest-posttest improvement in the “other” race and ethnicity stratum was 66.0 m (95% CI 49.9-199.5), suggesting intervention efficacy. The improvement for non-Hispanic White participants was not significant (median –17.2 m, 95% CI −33.8 to 44.7). The DiD of 44.0 m (95% CI 19.2-164.2; *P*=.004; adjusted *P*=.048) showed an association between walk distance and race and ethnicity, suggesting potential effect modification.

We observed significant pretest-posttest improvements in the stratum of talking to or seeing family and friends 3 or more times a week (median change 46.4 m, 95% CI 17.2-69.4); the other stratum, talking to or seeing family and friends less than 3 times a week, had only 2 participants, and it was not tenable to construct a CI at the 95% confidence level. Although the associated DiD of 77.0 m (95% CI −45.7 to 170.9; *P*=.20) was performed inherited uncertainties from the stratum of less than 3 times a week made it difficult to assure the true value of the DiD.

For educational attainment, we observed statistically significant pretest-posttest median changes in the stratum of college or higher educational level (median change 48.8 m, 95% CI 28.0-61.4) compared with a smaller and nonsignificant median change (29.6, 95% CI −28.1 to 114.1) in the stratum of lower than college educational level. The DiD of –16.5 m (95% CI −76.9 to 33.6; *P*=.70) was not significant.

There was statistical evidence of MI efficacy in 3 additional SDOH strata, namely, the “other” (widowed, single, or divorced) stratum of marital status, having no unmet needs, and living with family. DiD analyses were not significant for these 3 SDOH. Uncertainties for the first 2 were higher due to a size of 2 for the complementary stratum.

**Table 1:** Within-stratum median changes in 6-minute walk distance from baseline to 3 months after the motivational interviewing intervention and between-stratum difference-in-differences (DiD) by social determinants of health.

Variables			Participants, n		Baseline 6-min walk distance (m), median (IQR)		Postintervention 6-min walk distance (m), median (IQR)		Within-stratum median pretest-posttest change (m; 95% CI)		Between-stratum difference				
											DiD (m; 95% CI)			*P* value^a^	
**Sex**												−2.5 (−44.8 to 138.1)			.90 (0.95)
	Male	5		339.5 (243.8-359.5)		367.5 (335.3-379.0)		43.2 (−15.2 to 199.5)							
	Female	9		277.8 (213.4-304.4)		327.7 (218.4-350.5)		40.5 (−27.0 to 61.4)							
**Ethnicity and race**												*44.0 (19.2 to 164.2)* ^b^			.004 (0.05)
	Others	5		243.8 (179.5-277.8)		347.7 (302.2-350.5)		*66.0 (49.9 to 199.5)*							
	Non-Hispanic White	9		309.7 (224.3-350.5)		335.3 (263.5-365.8)		17.2 (−33.8 to 44.7)							
**Marital status**												−104.8 (−226.5 to 81.3)			.79 (0.95)
	Others	12		287.7 (221.5-317.1)		337.7 (252.2-355.1)		*40.4 (0.5 to 56.0)*							
	Married	2		265.0 (222.3-307.8)		357.2 (346.2-368.1)		92.1^c^							
**Educational attainment**												−16.5 (−76.9 to 33.6)			.70 (0.95)
	Below college	9		277.8 (179.5-350.5)		335.3 (218.4-351.5)		29.6 (−28.1 to 114.1)							
	College or higher	5		297.7 (243.8-304.4)		347.7 (302.2-365.8)		*48.8 (28.0 to 61.4)*							
**Household income (US $)**												10.4 (−126.9 to 65.2)			.62 (0.95)
	<30,000	7		243.8 (218.8-287.7)		302.2 (189.3-349.1)		47.3 (−29.5 to 65.5)							
	≥30,000	7		339.5 (244.6-352.6)		351.5 (331.5-373.3)		31.8 (−15.2 to 120.7)							
**Total ACEs^d^**												−2.9 (−81.3 to 42.3)			.95 (0.95)
	0-1	6		287.7 (252.3-337.3)		331.5 (308.6-344.6)		28.8 (−27.0 to 72.7)							
	2-8	8		258.9 (172.7-317.1)		358.6 (192.6-370.4)		44.9 (−33.8 to 120.7)							
**Military service**												12.6 (−128.6 to 171.5)			.51 (0.95)
	No	11		277.8 (218.8-330.1)		335.3 (282.8-351.0)		41.2 (−16.1 to 72.7)							
	Yes	2		185.0 (107.7-262.3)		214.2 (137.6-290.9)		29.2^c^							
**Living with family**												−37.6 (−148.1 to 6.7)			.08 (0.52)
	No	8		324.6 (269.3-351.5)		342.9 (321.3-355.5)		18.6 (−35.1 to 57.3)							
	Yes	6		201.9 (159.2-279.3)		305.5 (229.7-361.2)		*55.7 (30.5 to 199.5)*							
**Employed**												−6.4 (−65.2 to 133.5)			.85 (0.95)
	Yes	6		330.1 (305.7-353.6)		358.7 (339.3-375.7)		35.3 (−27.0 to 199.5)							
	No	8		234.0 (198.1-282.8)		282.8 (192.6-348.4)		44.5 (−29.5 to 65.5)							
**Health insurance status**												—^e^			.42 (0.95)^f^
	Uninsured	2		307.0 (305.7-308.4)		358.6 (355.1-362.2)		51.6^c^							
	Private	3		277.8 (228.7-316.2)		350.5 (339.1-364.8)		79.5^c^							
	Public	9		243.8 (218.4-318.6)		302.2 (218.4-347.7)		30.5 (−29.5 to 52.6)							
**Any unmet needs**												−40.6 (−170.7 to 71.2)			.20 (0.65)
	Yes	2		361.0 (357.8-364.3)		362.0 (344.8-379.1)		0.90^c^							
	None	12		260.8 (204.9-305.7)		341.5 (252.2-355.1)		*46.5 (7.6 to 67.1)*							
**Seeing or talking to family and friends**												77.0 (−45.7 to 170.9)			.20 (0.65)
	<3 times per wk	2		121.9		88.1		33.8^c^							
	≥3 times per wk	12		301.1		349.1		*46.4 (17.2 to 69.4)*							
**Stress**												—^e^			.68 (0.95)^f^
	Very much	4		223.5 (213.4-297.7)		363.3 (289.5-383.3)		44.6^c^							
	Some	5		260.8 (175.3-297.7)		327.7 (218.4-350.5)		48.2 (−27.0 to 72.7)							
	No	5		309.7 (243.8-339.5)		335.3 (302.2-351.5)		34.9 (−15.2 to 58.3)							

^a^False discovery rate (Benjamini-Hochberg)–adjusted *P* values in parentheses.

^b^Italics indicate statistically significant differences in median changes.

^c^We could not obtain a CI at the 95% level due to the small stratum size.

^d^ACE: adverse childhood experience.

^e^For SDOH with more than two strata, we do not report pairwise DiDs and their CI. We only report *P* value.

^f^Kruskal-Wallis test.

### Secondary Outcome: Body Weight

We observed statistical evidence of intervention efficacy in weight loss in the following SDOH strata: other ethnicity or race group, other marital status (widowed, divorced, or single), college or higher educational attainment, no unmet needs, and seeing and talking to family and friends 3 or more times a week (Table 2). These were the strata where we also observed efficacy evidence in 6-minute walk distance. Additionally, we observed efficacy evidence in the following SDOH strata that were not observed for 6-minute walk distance: male sex, household income of US $30,000 or higher, no military service, being employed (full or part time), and no stress. No statistically significant DiD was observed in any SDOH for weight loss. The statistical evidence of intervention efficacy in the 5 SDOH strata that were common between both the primary and secondary outcomes indicated a good degree of reproducibility of intervention efficacy, suggesting an underlying likelihood of intervention heterogeneity.

**Table 2:** Median weight loss from baseline to 3 months after the motivational interviewing intervention and between-stratum difference-in-differences by social determinants of health.

Variables		Participants, n	Baseline weight loss (lb), median (IQR)	Postintervention weight loss (lb), median (IQR)	Median pretest-posttest difference (lb; 95% CI)	Difference-in-differences between strata (lb; 95% CI)		*P* value^a^	
**Sex**							0.0 (−18.4 to 14.8)		>.99 (1.0)
	Male	5	256.0 (191.8-270.0)	244.2 (181.8-245.0)	*−10.0 (−25.8 to −3.0)^b^*				
	Female	9	250.4 (207.0-320.0)	243.0 (211.0-271.1)	−10.1 (−28.2 to 6.0)				
**Ethnicity and race**							−1.8 (−17.4 to 14.8)		.68 (1.0)
	Others	5	194.2 (181.6-256.0)	185.0 (172.0-245.0)	*−11.0 (−24.8 to −3.0)*				
	Non-Hispanic White	9	270.0 (237.4-280.6)	244.2 (230.0-271.1)	−8.7 (−28.1 to 6.5)				
**Marital status**							−3.0 (−37.9 to 27.4)		.84 (1.0)
	Others	12	243.9 (193.6-290.5)	236.5 (184.2-265.0)	*−10.3 (−22.4 to −1.7)*				
	Married	2	267.2 (261.6-272.8)	259.5 (252.3-266.8)	−7.7^c^				
**Educational attainment**							1.8 (−15.6 to 21.4)		.83 (1.0)
	Below college	9	256.0 (207.0-320.0)	245.0 (211.0-274.0)	−9.2 (−28.2 to 6.0)				
	College or higher	5	237.4 (194.2-270.0)	230.0 (185.0-244.2)	*−12.5 (−25.8 to −3.0)*				
**Household income (US $)**							13.8 (−1.0 to 33.0)		.09 (0.59)
	<30,000	7	207.0 (187.9-259.0)	211.0 (178.5-246.5)	−5.7 (−13.4 to 10.0)				
	≥30,000	7	270.0 (253.2-299.2)	245.0 (243.6-272.6)	*−17.0 (−36.9 to −7.2)*				
**Total ACEs^d^**							7.5 (−11.4 to 21.4)		.19 (0.62)
	0-1	6	215.8 (184.8-247.2)	207.5 (175.3-239.8)	−7.4^c^				
	2-8	8	275.3 (243.8-327.5)	254.0 (235.9-284.9)	−14.1 (−33.3 to 6.0)				
**Military service**							−21.1 (−71.9 to 29.8)		.91 (1.0)
	No	11	237.4 (193.0-267.2)	230.0 (183.4-258.1)	*−9.2 (−24.8 to −3.7)*				
	Yes	2	310.0 (290.0-330.0)	308.6 (276.4-340.8)	−1.4^c^				
**Living with family**							0.50 (−30.4 to 13.8)		.93 (1.0)
	No	8	228.7 (189.3-272.1)	227.0 (179.4-250.9)	*−9.6 (−29.5 to −1.7)*				
	Yes	6	268.3 (242.1-332.7)	254.0 (233.8-310.4)	−10.1 (−24.8 to 23.0)				
**Employed**							−5.5 (−33.0 to 8.2)		.39 (1.0)
	Yes	6	267.2 (251.8-280.1)	254.0 (243.5-269.1)	*−11.0 (−48.9 to −4.4)*				
	No	8	222.2 (191.1-290.0)	220.5 (181.8-264.7)	−7.9 (−18.4 to 7.8)				
**Health insurance status**							—^e^		.15 (0.62)^f^
	Uninsured	2	300.3 (290.5-310.2)	267.1 (265.0-269.1)	−33.3^c^				
	Public	9	207.0 (190.0-274.2)	230.0 (185.0-274.0)	−6.8 (−17.4 to 6.9)				
	Private	3	250.4 (216.0-253.2)	243.0 (206.8-244.0)	−9.5^c^				
**Any unmet needs**							1.0 (−30.4 to 38.9)		.84 (1.0)
	Yes	2	221.1 (206.5-235.8)	212.4 (197.1-227.7)	−8.7^c^				
	None	12	263.0 (203.8-290.5)	244.6 (204.5-271.8)	*−10.9 (−22.4 to −1.4)*				
**Seeing and talking to family and friends**							29.3 (8.4 to 52.9)		.02 (0.26)
	<3 times per wk	2	278.5 (242.8-314.3)	292.0 (251.5-332.5)	13.5^c^				
	≥3 times per wk	12	253.2 (193.6-279.0)	243.6 (184.2-265.0)	*−12.5 (−25.3 to −7.4)*				
**Stress**							—^e^		.90 (1.0)^f^
	Very much	4	194.2 (191.8-207.0)	198.0 (184.2-219.5)	−9.4^c^				
	Somewhat	5	265.5 (198.8-332.7)	263.0 (243.0-326.2)	−11.0 (−24.8 to 23.0)				
	No	5	270.0 (237.4-278.4)	244.2 (230.0-271.1)	*−15.1 (−48.9 to −3.0)*				

^a^False discovery rate (Benjamini-Hochberg)–adjusted *P* values in parentheses.

^b^Italics indicate statistically significant differences in median changes.

^c^We could not obtain a CI at the 95% confidence level due to the small stratum size.

^d^ACE*:* adverse childhood experience.

^e^For SDOH with more than two strata, we do not report pairwise DiDs and their CI. We only report *P* value.

^f^Kruskal-Wallis test.

## Discussion

Collins et al [12] reported significantly greater improvements in 6-minute walk distance and weight loss among patients with PAD receiving the MI intervention than among those receiving the smartphone app. The size of improvement in 6-minute walk distance exceeded the threshold of large clinically meaningful changes (20 m) [11]. This large-size improvement not only enhanced the underlying statistical power of the study but also made the current exploration of intervention heterogeneity feasible and relevant. To our knowledge there is a persistent knowledge gap regarding the extent to which SDOH would modify intervention effects and result in intervention heterogeneity within the context of PAD. Collins et al [12] did not explore this either. Studies have shown that declines in 6-minute walk distance and general physical function among patients with PAD are associated with SDOH such as race, socioeconomic status (SES), and educational attainment [25]. Therefore, evidence of these SDOH being potential intervention effect modifiers is crucial in designing studies to target modifiable risk factors and maximize benefits.

Smaller stratum sizes made interpretation of the results of within-stratum improvement (intervention efficacy) and DiD challenging. We noted a very high degree of consistency in our results across the 2 outcomes and all SDOH. Except for the stratum of having unmet needs (n=2), with a median improvement of 1 m in 6-minute walk distance, improvements ranged from 19 to 80 m, near or greater than the large clinical threshold of 20 m. In weight loss, except for the strata of seeing or talking to family and friends less than 3 times a week (n=2) and military service (n=2), weight loss ranged between 6 and 33 lb across the rest of the SDOH strata. Such consistency is strong evidence that the likelihood of spurious findings was small.

We noted that a group size below 5 was likely to result in a breakdown in tests and CIs, giving rise to further uncertainty in DiD. On the other hand, the exceptionally large effect sizes that we observed in many SDOH strata compensated for the statistical power associated with smaller sizes.

We found an association between race and ethnicity and 6-minute walk distance: patients of “other” race and ethnicity (n=5) exhibited a significant and greater median improvement (66.0 m, 95% CI 49.9-199.5) in 6-minute walk distance than non-Hispanic White patients (n=9; 17.2 m, 95% CI −33.8 to 44.7). The nonsignificant improvement in non-Hispanic White patients could be the result of limited statistical power and, subsequently, may make the associated DiD less stable. We note that most of this “other” group actually comprised Black patients. This finding is relevant because it suggests that the MI intervention might have helped patients of “other” race and ethnicity overcome barriers to physical activity that minority patients with PAD in general, and Black patients in particular, are more likely to encounter. Rucker-Whitaker et al [26] reported that Black patients with PAD, who were also less likely to have a college-level educational attainment and more likely to have lower SES, generally performed more poorly in the 6-minute walk test. Thus, interventions may target barriers to physical activity that are more prevalent among people with lower educational attainment and SES. The observation that the patients with higher educational attainment (college degree and higher) exhibited significant and sizable improvements in both 6-minute walk distance and weight loss suggests and supports a plausible health literacy pathway in which an intervention could reduce knowledge gaps in physical activity and healthy diet in minority patients with PAD, thereby mediating and partially compensating for their relatively lower educational attainment and SES [27]. The statistical evidence of significant improvements in both 6-minute walk distance and weight loss among participants who talked to or saw family members and friends 3 times or more a week indicated another plausible pathway of mediation parallel to health literacy—improving social support [28]. Together, our association findings have generated a broad working hypothesis: interventions that modify and mitigate barriers that patients with PAD likely face due to their SDOH could have greater impacts and reduce PAD disparities.

The smaller stratum sizes were a major limitation of this study, which gave rise to uncertainties regarding intervention efficacy within a stratum (when improvements were not statistically significant) and, subsequently, DiD estimation between strata for effect modification or intervention heterogeneity. We provided illustrations in several scenarios and advocated for cautionary interpretation. In particular, we demonstrated in this study that the consistency in results across SDOH strata and outcomes was an important source of confidence.

In conclusion, this exploratory investigation found that race and ethnicity were significantly associated with the intervention effect in terms of 6-minute walk distance. It also found significant evidence of intervention efficacy in some strata related to educational attainment and social support (frequency of seeing and talking to family and friends), indicators of 2 well-established mediation pathways to health improvement through health literacy and social support. This evidence is consistent with the literature on the associations between SDOH and general functions in patients with PAD and health pathways. Given the dearth of knowledge and evidence on SDOH-related disparities in PAD interventions, this pilot study generated useful hypotheses regarding intervention heterogeneity alongside SDOH by effectively using limited data. These hypotheses regarding the mediation effects of SDOH can be verified with larger stratum sizes. Future studies on PAD interventions should incorporate intervention heterogeneity in study design to target modifiable pathways and maximize potential benefits for socially disadvantaged patients with PAD. With systemic measurements and study of SDOH [29], the knowledge to be gained in this space will likely have significant impact on intervention design, implementation, and adaptation for PAD and beyond.

## Data Availability

The dataset generated or analyzed during this study are available from the authors on reasonable request.

## Appendix

Multimedia Appendix 1 Design, implementation, and evaluation of the original trial [8].

Multimedia Appendix 2 CONSORT checklist.

## Abbreviations

ACE: adverse childhood experience
DiD: difference-in-differences
MI: motivational interviewing
PAD: peripheral artery disease
PRAPARE: Protocol for Responding to and Assessing Patients’ Assets, Risks, and Experiences
SDOH: social determinants of health
SES: socioeconomic status
