# Supplementary material for: The Moderating Effects of Race and Ethnicity on a Motivational Interviewing Intervention for Improving Physical Health Among Patients With Peripheral Artery Disease: Secondary Analysis of a Pilot Randomized Trial
Source: JMIR Form Res. 2026 Oct 15;10:e82926. doi: 10.2196/82926 (PMC13720403; doi:10.2196/82926)
Supplement: Supplementary file 1 [file formative_v10i1e82926_app2.pdf]

CONSORT-EHEALTH Checklist V1.6.2 Report	Manuscript Number	30295

(based on CONSORT-EHEALTH V1.6), available at [http://tinyurl.com/consort-ehealth-v1-6].

Date completed

1/24/2022 19:28:12

by

Tracie Collins

Use of a Smartphone Application versus Motivational Interviewing to Increase Walking Distance and Weight Loss in Overweight/Obese Adults with Peripheral Artery Disease: Pilot Randomized Trial

TITLE

1a-i) Identify the mode of delivery in the title

Use of a Smartphone Application versus Motivational Interviewing to Increase Walking Distance and Weight Loss in Overweight/Obese Adults with Peripheral Artery Disease: A Pilot Randomized Trial

1a-ii) Non-web-based components or important co-interventions in title

1a-iii) Primary condition or target group in the title

"Use of a Smartphone Application versus Motivational Interviewing to Increase Walking Distance and Weight Loss in Overweight/Obese Adults with Peripheral Artery Disease: A Pilot Randomized Trial"

ABSTRACT

1b-i) Key features/functionalities/components of the intervention and comparator in the METHODS section of the ABSTRACT

"Patients were randomized into two groups: 1) MI, delivered through in-person and telephone counseling, or 2) App, a mobile smartphone application. Both interventions encouraged walking for exercise and healthy dietary habits (increasing fruits and vegetables as well as whole grains while reducing fat and sugary drinks)."

1b-ii) Level of human involvement in the METHODS section of the ABSTRACT

1b-iii) Open vs. closed, web-based (self-assessment) vs. face-to-face assessments in the METHODS section of the ABSTRACT

"We recruited study participants using a variety of modalities which included both the University of Kansas hospital system as well as unaffiliated health centers and community-based clinics. Area clinicians and administrators voluntarily distributed flyers and mailings to eligible patients."

1b-iv) RESULTS section in abstract must contain use data

"We randomized 29 participants with a mean age of 66.03 (SD 8.12) years. Twenty five participants completed the trial."

1b-v) CONCLUSIONS/DISCUSSION in abstract for negative trials

INTRODUCTION

2a-i) Problem and the type of system/solution

"Peripheral artery disease (PAD) is atherosclerosis of the of the abdominal aorta and arteries of the lower extremities that causes stenosis or occlusion.[1] Patients with PAD are at increased risk for cardiovascular events and functional decline.[2-5] As such, treatment for PAD has focused not only on risk factor modification but also on improving walking distance.[6-10]

Community-based walking therapy is an effective non-invasive treatment option for PAD. [11-13] Motivating adherence to community-based walking therapy can be achieved through traditional, in-person approaches. Alternatively, motivating adherence can be achieved through mobile health or counseling. [8, 9]. We explored the potential efficacy of a smartphone application (App) versus a well-known counseling approach, motivational interviewing, to improve walking distance and promote weight loss among overweight/obese adults with PAD.

Although walking therapy is an important component of improving outcomes in patients with PAD, less is known about the efficacy of dietary interventions for disease management—in particular, to promote weight loss in overweight/obese patients with PAD.

Our pilot study is novel insofar as it focuses on both walking therapy combined with a dietary intervention. We delivered this combined intervention using two approaches: a smartphone application (App) versus motivational interviewing (MI); MI was delivered both in-person and by phone. The App offers an innovative approach to behavior change for patients with PAD and the use of MI builds upon are prior work involving patients with PAD."

2a-ii) Scientific background, rationale: What is known about the (type of) system

"Peripheral artery disease (PAD) is atherosclerosis of the of the abdominal aorta and arteries of the lower extremities that causes stenosis or occlusion.[1] Patients with PAD are at increased risk for cardiovascular events and functional decline.[2-5] As such, treatment for PAD has focused not only on risk factor modification but also on improving walking distance.[6-10]

Community-based walking therapy is an effective non-invasive treatment option for PAD. [11-13] Motivating adherence to community-based walking therapy can be achieved through traditional, in-person approaches. Alternatively, motivating adherence can be achieved through mobile health or counseling. [8, 9]. We explored the potential efficacy of a smartphone application (App) versus a well-known counseling approach, motivational interviewing, to improve walking distance and promote weight loss among overweight/obese adults with PAD.

Although walking therapy is an important component of improving outcomes in patients with PAD, less is known about the efficacy of dietary interventions for disease management—in particular, to promote weight loss in overweight/obese patients with PAD.

Our pilot study is novel insofar as it focuses on both walking therapy combined with a dietary intervention. We delivered this combined intervention using two approaches: a smartphone application (App) versus motivational interviewing (MI); MI was delivered both in-person and by phone. The App offers an innovative approach to behavior change for patients with PAD and the use of MI builds upon are prior work involving patients with PAD."

Does your paper address CONSORT subitem 2b?

"In this pilot, we explored the potential efficacy of our App vs. MI to increase walking distance and promote weight loss among overweight/obese adults with PAD."

METHODS

3a) CONSORT: Description of trial design (such as parallel, factorial) including allocation ratio

"We conducted a 3-month, 2-arm randomized pilot study at the University of Kansas."

3b) CONSORT: Important changes to methods after trial commencement (such as eligibility criteria), with reasons

There were no changes to the methods after trial commencement.

3b-i) Bug fixes, Downtimes, Content Changes

4a) CONSORT: Eligibility criteria for participants

"Inclusion criteria were age 50 years or older, overweight/obese (BMI > 27), and symptomatic PAD – leg symptoms were captured by a survey and PAD was confirmed with the use of the ankle-brachial index (ABI) which is the ratio of the systolic blood pressure in the ankle to that in the arm. Our cut-point for the ABI was <0.9.

Individuals were excluded if they demonstrated at least one of the following conditions: intolerance to fruits, vegetables, fiber, and/or a low-fat diet, restricted water intake, pregnant, prior major ischemia or critical leg ischemia, use of 24-hour supplemental oxygen, heart attack within the last 3 months, inability to walk for exercise, or currently walking at least 3 days per week for at least 30 minutes each day."

4a-i) Computer / Internet literacy

4a-ii) Open vs. closed, web-based vs. face-to-face assessments:

Participants were recruited from clinical settings.

4a-iii) Information giving during recruitment

4b) CONSORT: Settings and locations where the data were collected

This information was included in the manuscript.

4b-i) Report if outcomes were (self-)assessed through online questionnaires

The manuscript provides language that outcomes were obtained by a blinded assessor.

4b-ii) Report how institutional affiliations are displayed

5) CONSORT: Describe the interventions for each group with sufficient details to allow replication, including how and when they were actually administered

5-i) Mention names, credential, affiliations of the developers, sponsors, and owners

The paper addresses subitem 5-i

**5-ii) Describe the history/development process**		
This information is included in the manuscript.		
**5-iii) Revisions and updating**		
**5-iv) Quality assurance methods**		
**5-v) Ensure replicability by publishing the source code, and/or providing screenshots/screen-capture video, and/or providing flowcharts of the algorithms used**		
**5-vi) Digital preservation**		
**5-vii) Access**		
"On the first day of the study, patients installed the App, and it prompted them to enter their demographic, health, and identity information. The App then presented patients with six possible nutritional changes that they could choose to address during the first two-week cycle (Figure 1). Once participants chose one of the six dietary modifications, they were prompted to perform a self-assessment; the App would then adjust goals according to patients' responses.<br><br>We made explicit efforts to ensure that patients would not have trouble installing, using, or interacting with the application. Several safeguards were installed to provide patients with continuous support and resources."		
**5-viii) Mode of delivery, features/functionalities/components of the intervention and comparator, and the theoretical framework**		
"The App was designed for use with Android OS smartphones—patients without this type of phone received it as part of the study. We tailored nutritional content for clarity and lower health literacy levels. We designed the mobile app to allow users to input walking plans, track walking intervals, and record episodic pain. We designed in-app notifications to encourage patient use as well as diet and exercise management.<br><br>The App was completely automated and adaptive to patient use. The study team was available to troubleshoot and answer questions as needed. Safeguards permitted recovery of previously saved data in the event of accidental malfunction. Participant data was encrypted and saved locally to the phone. Once initialized, the App sent secure, de-personalized data to an online backup server via the internet."		
**5-ix) Describe use parameters**		
"On the first day of the study, patients installed the App, and it prompted them to enter their demographic, health, and identity information. The App then presented patients with six possible nutritional changes that they could choose to address during the first two-week cycle (Figure 1). Once participants chose one of the six dietary modifications, they were prompted to perform a self-assessment; the App would then adjust goals according to patients' responses.<br><br>We made explicit efforts to ensure that patients would not have trouble installing, using, or interacting with the application. Several safeguards were installed to provide patients with continuous support and resources. The App provided extensive exercise tracking (Figure 1) as well as a comprehensive reporting menu with information about patient progress (Figure 1). Automatic reminders prompted participants to engage with the App and to select their next PAD intervention cycle at two-week intervals."		
**5-x) Clarify the level of human involvement**		
**5-xi) Report any prompts/reminders used**		
"Automatic reminders prompted participants to engage with the App and to select their next PAD intervention cycle at two-week intervals."		
**5-xii) Describe any co-interventions (incl. training/support)**		
There were no co-interventions with the app.		
**6a) CONSORT: Completely defined pre-specified primary and secondary outcome measures, including how and when they were assessed**		
This information is included in the manuscript abstract and body.		
**6a-i) Online questionnaires: describe if they were validated for online use and apply CHERRIES items to describe how the questionnaires were designed/deployed**		
**6a-ii) Describe whether and how "use" (including intensity of use/dosage) was defined/measured/monitored**		
**6a-iii) Describe whether, how, and when qualitative feedback from participants was obtained**		
We administered an exit interview.		
**6b) CONSORT: Any changes to trial outcomes after the trial commenced, with reasons**		
This information was included in the manuscript.		
**7a) CONSORT: How sample size was determined**		
**7a-i) Describe whether and how expected attrition was taken into account when calculating the sample size**		
We provided a power calculation for attrition.		
**7b) CONSORT: When applicable, explanation of any interim analyses and stopping guidelines**		
This information is included in the manuscript abstract and body.		
**8a) CONSORT: Method used to generate the random allocation sequence**		
"We used a random number generator to blindly assign patients to one of the two groups."		
**8b) CONSORT: Type of randomisation; details of any restriction (such as blocking and block size)**		
This information is provided in the manuscript.		
**9) CONSORT: Mechanism used to implement the random allocation sequence (such as sequentially numbered containers), describing any steps taken to conceal the sequence until interventions were assigned**		
This information is provided in the manuscript		
**10) CONSORT: Who generated the random allocation sequence, who enrolled participants, and who assigned participants to interventions**		
"We used a random number generator to blindly assign patients to one of the two groups."		
**11a) CONSORT: Blinding - If done, who was blinded after assignment to interventions (for example, participants, care providers, those assessing outcomes) and how**		
**11a-i) Specify who was blinded, and who wasn't**		
The outcomes assessor was blinded.		
**11a-ii) Discuss e.g., whether participants knew which intervention was the "intervention of interest" and which one was the "comparator"**		
**11b) CONSORT: If relevant, description of the similarity of interventions**		
The manuscript describes similarities between the theoretical framework for motivational interviewing and the smartphone app.		
**12a) CONSORT: Statistical methods used to compare groups for primary and secondary outcomes**		
"We used Wilcoxon rank-sum test to analyze the primary outcome of change in 6-minute walking distance at three months within the MI and App groups, and to compare the changes between the groups with adjustment for baseline (difference in difference). We used this same method for other outcomes. For comparing participants' baseline characteristics between the MI and App groups, we used Wilcoxon test for continuous measurements and Fisher's test for dichotomous measurements. We use R version 4.03 (R Foundation for Statistical Computing) for all data analyses."		
**12a-i) Imputation techniques to deal with attrition / missing values**		
For such a small sample, we did not use imputation methods.		
**12b) CONSORT: Methods for additional analyses, such as subgroup analyses and adjusted analyses**		
There were no subgroup analyses for this pilot.		
RESULTS		
**13a) CONSORT: For each group, the numbers of participants who were randomly assigned, received intended treatment, and were analysed for the primary outcome**		
This information is provided in the manuscript.		
**13b) CONSORT: For each group, losses and exclusions after randomisation, together with reasons**		
This information is provided in the manuscript.		
**13b-i) Attrition diagram**		
This information is available.		
**14a) CONSORT: Dates defining the periods of recruitment and follow-up**		
This information is included in the manuscript.		
**14a-i) Indicate if critical "secular events" fell into the study period**		

**14b) CONSORT: Why the trial ended or was stopped (early)**		
The trial was not terminated early.		
**15) CONSORT: A table showing baseline demographic and clinical characteristics for each group**		
This information is available in the manuscript as a table and within the text.		
**15-i) Report demographics associated with digital divide issues**		
This information is provided in the manuscript		
**16a) CONSORT: For each group, number of participants (denominator) included in each analysis and whether the analysis was by original assigned groups**		
**16-i) Report multiple "denominators" and provide definitions**		
The analysis was based on intention to treat.		
**16-ii) Primary analysis should be intent-to-treat**		
**17a) CONSORT: For each primary and secondary outcome, results for each group, and the estimated effect size and its precision (such as 95% confidence interval)**		
This information is provided in the manuscript.		
**17a-i) Presentation of process outcomes such as metrics of use and intensity of use**		
**17b) CONSORT: For binary outcomes, presentation of both absolute and relative effect sizes is recommended**		
There were no binary outcomes.		
**18) CONSORT: Results of any other analyses performed, including subgroup analyses and adjusted analyses, distinguishing pre-specified from exploratory**		
There were no subgroup or exploratory analyses.		
**18-i) Subgroup analysis of comparing only users**		
**19) CONSORT: All important harms or unintended effects in each group**		
This was addressed in the manuscript.		
**19-i) Include privacy breaches, technical problems**		
**19-ii) Include qualitative feedback from participants or observations from staff/researchers**		
There was an exit interview but there was limited qualitative feedback.		
DISCUSSION		
**20) CONSORT: Trial limitations, addressing sources of potential bias, imprecision, multiplicity of analyses**		
**20-i) Typical limitations in ehealth trials**		
This information is addressed in the manuscript.		
**21) CONSORT: Generalisability (external validity, applicability) of the trial findings**		
**21-i) Generalizability to other populations**		
This was a pilot with some evidence to support the use of the app in patients with peripheral artery disease but there is limited generalizability given the small sample size.		
**21-ii) Discuss if there were elements in the RCT that would be different in a routine application setting**		
**22) CONSORT: Interpretation consistent with results, balancing benefits and harms, and considering other relevant evidence**		
**22-i) Restate study questions and summarize the answers suggested by the data, starting with primary outcomes and process outcomes (use)**		
The paper addresses subitem 22-i.		
**22-ii) Highlight unanswered new questions, suggest future research**		
Other information		
**23) CONSORT: Registration number and name of trial registry**		
"The clinical trial registration for this study is NCT03694652 (https://clinicaltrials.gov/)."		
**24) CONSORT: Where the full trial protocol can be accessed, if available**		
The full trial protocol can be accessed upon request to the primary author.		
**25) CONSORT: Sources of funding and other support (such as supply of drugs), role of funders**		
"Research reported in this publication was supported by the National Heart, Lung, And Blood Institute of the National Institute of Health under Award Number 1R56HL138244-01. The content is solely the responsibility of the authors and does not necessarily represent the official views of the National Institutes of Health."		
**X26-i) Comment on ethics committee approval**		
We include IRB approval in the manuscript.		
**x26-ii) Outline informed consent procedures**		
This information is in the manuscript.		
**X26-iii) Safety and security procedures**		
Research staff were available to answer any questions and to review reported adverse outcomes.		
**X27-i) State the relation of the study team towards the system being evaluated**		
