# Supplementary material for: The Moderating Effects of Race and Ethnicity on a Motivational Interviewing Intervention for Improving Physical Health Among Patients With Peripheral Artery Disease: Secondary Analysis of a Pilot Randomized Trial
Source: JMIR Form Res. 2026 Oct 15;10:e82926. doi: 10.2196/82926 (PMC13720403; doi:10.2196/82926)
Supplement: Supplementary file 2 [file formative_v10i1e82926_app1.docx]

**Supplement: Design, implementation, and evaluation of the original trial (Collins et al 2021).**

**Study Design**

We conducted a two-arm, randomized pilot trial comparing a smartphone application (App) with motivational interviewing (MI) among overweight or obese adults with symptomatic peripheral artery disease (PAD). The intervention period was three months. Participants were randomized in a 1:1 ratio to either MI or App following completion of baseline assessments. The primary objective was to evaluate preliminary efficacy on walking distance and weight loss and to assess feasibility of intervention delivery.

**Participant Screening, Enrollment, and Retention**

A total of 409 individuals were assessed for eligibility. Of these, 380 were excluded for reasons including ineligibility based on ABI criteria, claudication criteria, BMI criteria, age, inability to exercise safely, dietary restrictions, lack of Android phone access, participation in another study, or withdrawal of interest. Twenty-nine participants were randomized, with 16 assigned to MI and 13 assigned to the App intervention. Twenty-five participants completed the three-month follow-up assessment (14 MI and 11 App participants). Participant flow is summarized in the CONSORT diagram below

**Screened for eligibility** *(n = 409)*

## Enrollment

**Excluded** *(n = 380)*

- Did not meet ABI criteria *(n = 163)*
- Did not meet claudication criteria	*(n = 83)*
- Withdrew; lost interest *(n = 82)*
- Did not meet BMI criteria *(n = 5)*
- Did not meet age criteria *(n = 3)*
- No access to Android phone	*(n = 29)*
- Had limited ability to exercise due to health conditions *(n = 8)*
- Had dietary restrictions *(n = 4)*
- Currently enrolled in other studies *(n = 2)*
- Failed treadmill test *(n = 1)*

**Randomized** *(n = 29)*

**MI intervention** *(n = 16)*

**App intervention** *(n = 13)*

**Lost to 3-month follow-up** *(n= 2)*

## Follow-Up

## Analysis

## Allocation

**Lost to 3-month follow-up** *(n = 2)*

**Analyzed** *(n = 14)*

**Analyzed** *(n = 11)*

Figure 2. Consort Flow Diagram.

Figure S1. Consort Flow Chart

**Intervention Delivery**

Intervention fidelity was maintained through standardized procedures. Motivational interviewing was delivered by a trained counselor who received instruction from an experienced MI investigator, participated in practice sessions with standardized patients, and underwent ongoing supervision. The MI intervention consisted of one 60-minute in-person session followed by four structured 20-minute telephone sessions over three months.

Participants randomized to the App group installed the PAD Mobile App on an Android smartphone. They received automated nutrition and physical activity modules, reminders, goal-setting activities, self-monitoring features, and tailored feedback. Technical support was available as needed throughout the study period.

**Outcome Measures**

The primary outcome was change in six-minute walk distance from baseline to three months. Secondary outcomes included body weight, health-related quality of life measured with the VascuQol questionnaire, exercise behavior measured with the Stanford Exercise Behavior Survey, and dietary habits measured using the Fat-Related Diet Habits Questionnaire. All outcome assessments were conducted at baseline and three months using standardized procedures and validated instruments.

**Feasibility and Acceptability**

Feasibility was assessed through recruitment, retention, intervention completion, and participant engagement metrics. App engagement measures included frequency of App use, walking episodes recorded, completion of nutrition modules, responses to notifications, and completion of self-monitoring activities. Retention was defined as completion of the three-month follow-up assessment. Acceptability was assessed indirectly through intervention adherence and participant engagement; formal qualitative interviews and validated acceptability surveys were not collected in this pilot study.

Retention was high, with 25 of 29 randomized participants completing the three-month follow-up assessment (86%). Among App participants, engagement varied substantially. Participants generated 1,137 App interactions and recorded 355 walking episodes during the intervention period. Seventy-two percent of App participants initiated four or more nutrition modules, although only three participants completed all six modules. These findings demonstrate feasibility of recruitment, retention, and intervention delivery while identifying opportunities to improve user engagement with the nutritional components of the application.
